# Oncolytic adenovirus encoding a TGF-β inhibitor synergizes with PD-1 blockade to potentiate NK cell cytotoxicity against NSCLC

**DOI:** 10.3389/fimmu.2026.1759236

**Published:** 2026-02-25

**Authors:** Zhongqi Zhu, Chonghe Xu, Xiaoli Kong, Shulei Zhang, Shuping Lu, Shuo Zhang, Wei Xu, Qingmei Zhang, Mei Zhu

**Affiliations:** 1 Department of Clinical Laboratory, The Fourth Affiliated Hospital (Affiliated Chaohu Hospital) of Anhui Medical University, Chaohu, Anhui, China; 2 Beijing Anzhen Hospital, Capital Medical University, Beijing, China; 3 School of Basic Medical Sciences, Capital Medical University, Beijing, China; 4 Department of Blood Transfusion, The First Affiliated Hospital of Anhui Medical University, Hefei, Anhui, China; 5 Department of Anesthesiology, The Fourth Affiliated Hospital (Affiliated Chaohu Hospital) of Anhui Medical University, Chaohu, Anhui, China

**Keywords:** adoptive NK cell therapy, immunotherapy, non-small cell lung cancer, oncolytic adenovirus, TGF-β inhibitor

## Abstract

**Background:**

Immune checkpoint inhibitors (ICIs) are a frontline treatment for advanced non-small cell lung cancer (NSCLC), yet 80% of the patients exhibit resistance, creating an urgent need for novel therapeutic strategies. In this study, we investigated the synergistic efficacy of a triple-combination therapy comprising a PD-1 antibody, adoptive NK (natural killer) cells, and an oncolytic adenovirus Ad-anti-TGF-βRII (encoding a TGF-β inhibitor) in NSCLC xenograft mouse models.

**Methods:**

We investigated the combined effect of Ad-anti-TGF-βRII and NK cells on PD-1 antibody monotherapy using both *in vitro* cell experiments and the mouse model of NSCLC. Cytotoxicity assays, quantitative real-time PCR, and western blot analysis demonstrated that Ad-anti-TGF-βRII exhibited stronger tumor-killing activity compared to the control oncolytic adenovirus Ad-null. Furthermore, cytotoxicity assays and flow cytometry were employed to explore how the combination of Ad-anti-TGF-βRII and NK cells with PD-1 antibody promotes NK cell proliferation and activation, as well as the potent tumor-killing effect of the combination therapy. In the mouse model of NSCLC, the anti-tumor efficacy of the combination therapy was evaluated by monitoring tumor volume changes, hematoxylin and eosin (H&E) staining. The underlying mechanisms were further investigated using immunofluorescence, immunohistochemistry, quantitative real-time PCR, western blot, and flow cytometry.

**Results:**

The triple-combination therapy markedly inhibited tumor growth, augmented NK cell cytotoxicity and elevated the expression levels of perforin, granzyme B, and IFN-γ. Furthermore, it significantly increased lymphocyte recruitment and infiltration into tumor tissue. Comprehensive analysis demonstrated the favorable safety profile of this therapeutic regimen.

**Conclusions:**

Our findings suggest that the combination of a PD-1 antibody, NK cells, and the oncolytic adenovirus Ad-anti-TGF-βRII represents a promising therapeutic strategy for NSCLC by remodeling the tumor microenvironment (TME) to overcome ICI resistance.

## 1 Introduction

Lung cancer, with a one-year survival rate of 50% that drops to 19% at five years, remains the leading global cause of cancer-related mortality, causing more annual deaths than colorectal, breast, and prostate cancers combined (1–3). Non-small cell lung cancer (NSCLC), accounting for nearly 85% of all lung cancers, poses a significant global health challenge (4, 5). Despite advances in multidisciplinary treatments, NSCLC continues to exhibit high rates of recurrence and distant metastasis (6–8).

Immune checkpoint inhibitors (ICIs), particularly those reactivating endogenous antitumor responses by targeting the programmed cell death 1 (PD-1)/programmed death-ligand 1 (PD-L1) pathway, can disrupt tumor cells and reactivate effector T cells, prolonging survival in NSCLC patients (9–12). However, the overall efficacy of these agents remains limited, as approximately 80% of NSCLC patients develop primary or acquired resistance to ICI (13). Therefore, developing effective combination therapies to overcome ICI resistance in NSCLC is crucial.

Human natural killer (NK) cells are innate cytotoxic lymphoid cells that play a crucial role in eliminating virus-infected and tumor cells (14). Upon activation, they can rapidly eliminate abnormal cells through direct cytotoxicity and the secretion of pro-inflammatory cytokines like IFN-γ and TNF-α (15, 16). A key advantage of NK cells is their ability to mount anti-tumor responses without prior sensitization, acting as a critical first line of defense against malignancies (17, 18). Furthermore, accumulating evidence indicates that NK cells contribute to the therapeutic effects of PD-1/PD-L1 antibodies, particularly in tumors with low major histocompatibility complex (MHC) class I expression (19).

Despite progress in treating hematological malignancies, the efficacy of NK cell therapy in solid tumors remains limited. A major challenge for adoptive NK cell therapy in solid tumors is the immunosuppressive tumor microenvironment (TME) (20). NSCLC exemplifies an immunosuppressive tumor where immunosuppressive cells facilitate immune evasion and progression by suppressing T cell and NK cell antitumor functions, thereby contributing to ICI resistance (10).

Oncolytic viruses represent attractive immunotherapeutic agents due to their dual mechanism: selective tumor cell lysis and host immune system activation. They can effectively overcome immune tolerance within the TME by blocking immune checkpoints and converting immunosuppressive cells to a pro-inflammatory phenotype, thereby promoting infiltration of T cells and other immune cells, which modulates tumor immunogenicity and counteracts the immunosuppressive TME (21–23).

The engineered oncolytic adenovirus used encodes an inhibitor of transforming growth factor-β (TGF-β). TGF-β typically exerts inhibitory effects on immune cells. Released by tumor and stromal cells, TGF-β remodels the tumor microenvironment by promoting angiogenesis and immune escape, thereby facilitating tumor invasion and metastasis (24). Within the tumor immune microenvironment (TIME), TGF-β promotes tumor immune escape and metastasis by inducing NK cell dysfunction (25). This is mediated via the mothers against decapentaplegic homolog (SMAD) signaling pathway, where TGF-β-triggered SMAD2/3/4 complex formation translocates to the nucleus and suppresses the expression of critical antitumor effector molecules like IFN-γ and granzyme B, thereby crippling NK cell cytotoxicity (26). Therefore, inhibiting TGF-β can facilitate NK cell activation and help restore anti-tumor immunity (27). Previous studies have also indicated potential synergy between oncolytic adenoviruses and NK cells in cancer immunotherapy (20).

Here, we developed a genetically modified oncolytic adenovirus, Ad-anti-TGF-βRII, which encodes a TGF-β inhibitor. We hypothesized that this combination of direct oncolysis with local TGF-β blockade would remodel the immunosuppressive TME, enhance adoptively transferred NK cell function, and synergize with anti-PD-1 therapy to overcome ICI resistance in NSCLC.

## 2 Methods

### 2.1 Cell lines and cell culture

The human embryonic kidney cell line HEK293, the A549 lung adenocarcinoma cell line, and the human chronic myeloid leukemia cell line K562 were obtained from Procell Biotechnology Co., Ltd. (Wuhan, China). The A549 cell line was selected to establish the NSCLC model based on the findings of Yuan Cheng et al. (28), who demonstrated its utility in studying ICI resistance mediated by M2 macrophages. Cells were cultured in RPMI 1640 medium (Procell), which contains 10% fetal bovine serum (ExCell Bio) and 1% penicillin/streptomycin (Procell), and kept in a 37 °C incubator with 5% CO_2_. All cell lines were routinely tested for mycoplasma contamination.

### 2.2 Plasmid construction and cell transfection

The cDNA sequence encoding the human monoclonal antibody for TGF-βRII (GenBank Accession: BD094925.1) was cloned into the empty vector pDC316-mCMV-EGFP (FengHui, Hunan, China). The recombinant plasmid was transformed into E. coli 5-α competent cells (New England Biolabs, NEB, USA). Positive clones were selected with ampicillin (100 μg/mL), and subsequently confirmed by Sanger sequencing (performed by FengHui). Following plasmid preparation, A549 cells were seeded in six-well plates and transfected at approximately 60% confluence using Lipofectamine 3000 (Thermo Fisher Scientific, CA, USA) according to the manufacturer’s protocol. Cells were cultured for 48 h post-transfection. Transfection efficiency was assessed by fluorescence microscopy, and anti-TGF-βRII overexpression was confirmed by western blot analysis.

### 2.3 Preparation of the overexpression adenovirus clone

The adenoviral shuttle vector GV695 comprises the following elements in sequential order: CMV-MCS-3flag-TERTp-1A(CR2del)-E1B/19K. The linearized vector was generated by restriction endonuclease digestion (NEB, USA). The target gene fragment for TGF-β inhibitor overexpression was amplified by polymerase chain reaction (PCR). Homologous recombination sequences, designed to match the ends of the linearized vector, were incorporated into the 5’ ends of the amplification primers, ensuring the PCR product’s termini were identical to the vector ends. The linearized vector and the target gene PCR product were subjected to an *in vitro* recombination reaction, facilitating circularization. The recombinant product was directly transformed into competent cells. Single clones were selected for PCR screening, and positive clones were subjected to sequencing and sequence analysis. Validated recombinant plasmids were expanded and purified in high concentration for use in downstream adenovirus packaging.

### 2.4 Oncolytic adenovirus packaging

The genetically modified oncolytic adenovirus Ad5/3-E2F-d24 (viral backbone) was constructed as previously described (17). Expression of adenovirus E1A, essential for viral replication, is controlled by the human telomerase reverse transcriptase promoter. This design confers preferential replication in cells with elevated telomerase activity, notably cancer cells (29). A 24-base pair deletion (delta24) in the E1A gene, combined with modifications to the E2F promoter, restricts viral replication to tumor cells harboring abnormalities in the Rb/p16 pathway. The virus also carries a deletion in E1B/19K, enhancing tumor specificity. Ad-anti-TGF-βRII encodes a TGF-β inhibitor under the control of both the cytomegalovirus (CMV) promoter and the E1B promoter. Ad-null, an oncolytic adenovirus lacking transgenes, served as the control vector. HEK293 cells were co-transfected with the adenoviral shuttle plasmid (GV695) carrying the transgene and an auxiliary packaging plasmid devoid of adenoviral genomic regions (E1/E3 deletion). Recombinant, replication-selective adenovirus particles carrying the transgene were subsequently generated via Cre-loxP-mediated recombination.

### 2.5 Oncolytic adenovirus titer determination

Viral titers were determined using an endpoint dilution assay. HEK293 cells were seeded in a 96-well plate (1×10³ cells/well in 100 μL medium) 24 h prior to infection. The original adenovirus stock was diluted 1:100 in complete culture medium, and serial ten-fold dilutions (10^-1^ to 10^-10^) were then prepared. Medium was aspirated from the 96-well plate, and 90 μL of each virus dilution (10^-6^ to 10^-10^) was added to 10 wells per dilution. The 11th and 12th wells of each row received 90 μL of virus-free complete medium as negative controls. Plates were incubated at 37 °C with 5% CO_2_ for 10 days. Cytopathic effects (CPE) were assessed, and the number of CPE-positive wells per dilution recorded. The proportion of CPE-positive wells for each dilution was calculated to determine the virus titer using the formula: Virus titer = 10^(X + 0.8)^ (PFU/mL). In this context, X represents the cumulative sum of CPE positive rates for dilutions from 10–^1^ to 10^-13^. This formula is applicable only when there is: (i) no CPE or growth inhibition in negative controls, and (ii) CPE in all wells at the lowest dilution.

### 2.6 Expression of TGF-β inhibitor in oncolytic adenovirus-infected tumor cells

A549 cells were seeded in 6-well plates at a density of 7×10^5^ cells/well. After 24 h, cells were infected with either the therapeutic oncolytic adenovirus (Ad-anti-TGF-βRII) or the control adenovirus (Ad-null) at a multiplicity of infection (MOI) of 50. Six hours post-infection, the medium was replaced with fresh serum-free medium, and cells were cultured for an additional 48 h. Cells were then harvested, and total RNA was extracted using RNAiso reagent (Fastagen, Shanghai, China) according to the manufacturer’s protocol. Single-stranded cDNA was synthesized from the RNA using the PrimeScript RT reagent Kit (Takara, Shiga, Japan), and gene expression was then analyzed by quantitative real-time reverse transcription polymerase chain reaction (RT-qPCR) and western blotting.

### 2.7 Expansion of natural killer cells

Peripheral blood mononuclear cells (PBMCs) were isolated from healthy donor blood using Lymphoprep (Solarbio, Beijing, China) density gradient centrifugation. Isolated PBMCs were washed twice with 1× phosphate-buffered saline (PBS; Servicebio Technology Co., Ltd.). PBMCs were then resuspended in Lymphocyte Serum Free Medium (88-581-CM) supplemented with 10% FBS, 100 U/mL penicillin, 10 μg/mL streptomycin, 200 IU/mL recombinant human IL-2 (PeproTech, Rocky Hill, NJ, USA), and the NK cell expansion/activation agent (LYN000101; HangZhou LuYuan Biotechnology Co., Ltd.). This mixture was then cultured in a T75 culture flask. Additional NK cell expansion/activation agent was added on day 0 and day 7. Fresh complete medium was added every 2–3 days to maintain a cell density of 0.8-1.5×10^6^ cells/mL. After 14 days of expansion, NK cells were harvested. Only NK cell populations exhibiting >90% purity (as determined by flow cytometry) and confirmed cytotoxicity were used in subsequent experiments.

### 2.8 Cell viability and cytotoxicity assay

Cell viability was determined using the CCK-8 assay (GLPBIO, CA, USA) according to the manufacturer’s instructions. Briefly, A549 cells were plated at a density of 1×10^3^ cells/well into 96-well plates and allowed to adhere overnight. Following various treatments (e.g., oncolytic adenovirus infection, co-culture with NK cells, PD-1 antibody treatment, or exposure to conditioned supernatant), 10 μL of CCK-8 solution was added per well. Plates were incubated at 37 °C for 3 h, and the absorbance at 450 nm was measured using a microplate reader (Tecan, Männedorf, Switzerland). The mean absorbance of the control group was defined as 100% viability, and values from treated groups were normalized accordingly.


$$\text{Viability }(\%)=\frac{{\text{OD}}_{\text{treated}}-{\text{OD}}_{\text{blank}}}{{\text{OD}}_{\text{control}}-{\text{OD}}_{\text{blank}}}\times 100\%$$

$$\text{Cytotoxicity }(\%)=(1-\frac{{\text{OD}}_{\text{treated}}-{\text{OD}}_{\text{blank}}}{{\text{OD}}_{\text{control}}-{\text{OD}}_{\text{blank}}})\times 100\%$$

Four distinct CCK-8 experiments were conducted:

#### 2.8.1 Virus cytotoxicity assay

To quantify virus-mediated cytotoxicity, A549 cells were infected with Ad-anti-TGF-βRII at varying MOIs or with the control adenovirus, Ad-null.

#### 2.8.2 NK cell enhancement assay

To evaluate whether the TGF-β inhibitor-expressing oncolytic adenovirus enhances NK cell efficacy, expanded NK cells were co-cultured with A549 cells at a 1:10 effector-to-target (E:T) ratio, with or without PD-1 blocking antibody (Tislelizumab; GLPBIO). After 24 h, Ad-null or Ad-anti-TGF-βRII was added at MOI 100. Negative controls were included. The optimal antibody concentration (5 μg/mL) and incubation time (48 h) were determined prior to the main experiments (**Supplementary Figure 1**). This optimized regimen was then used in all subsequent combination therapy experiments.

#### 2.8.3 Supernatant effect on NK proliferation

NK cell culture medium was replaced with supernatant from oncolytic virus-infected A549 cells, and proliferation rates were assessed after 24 h.

#### 2.8.4 Combination therapy screening

A549 cells received five distinct treatments (e.g., Ad-anti-TGF-βRII, NK cells, PD-1 antibody, or combinations), followed by viability assessment.

### 2.9 Flow cytometry analysis

#### 2.9.1 Surface receptors expression on NK cells

To assess the impact of Ad-null and Ad-anti-TGF-βRII on NK cell receptors, supernatants from A549 cells infected with the oncolytic viruses were separately collected and used to treat NK cells for 48 h. The NK cells were then harvested and co-incubated with a CD335 (NKp46) Monoclonal Antibody (17-3359-41, Invitrogen, CA, USA) and a PE/Cyanine5 anti-human CD159a (NKG2A) Antibody (375111, Biolegend, CA, USA). Samples were analyzed on a flow cytometer (CytoFLEX) with appropriate compensation settings. Data were acquired using FlowJo v.10.8.1 software to analyze the expression levels of activating and inhibitory receptors on the surface of NK cells.

#### 2.9.2 Intracellular granzyme B and perforin in NK cells

NK cells were collected and stained with CD56-APC, CD16-APC, and CD3-PerCP at room temperature in the dark for 30 min. Cells were then fixed with fixative for 30 min, then permeabilized with permeabilizer for 20 min. Subsequently, cells were incubated with granzyme B-FITC and perforin-PE for 30 min. All antibodies and reagents for this assay were from Qingdao Raisecare Biological Technology Co., Ltd. The intracellular levels of granzyme B and perforin in NK cells were then quantified by flow cytometry.

#### 2.9.3 Cytokine secretion in co-culture systems

To investigate changes in cytokine secretion from A549 cells following treatment with a TGF-β inhibitor-encoding virus and NK cells, the cells were seeded in triplicate and treated with different combinations of Ad-anti-TGF-βRII, NK cells at a ratio of 1:10 (E:T), or a PD-1 antibody. After 72 h of co-culture, the supernatants were harvested and analyzed using the Cytometric Bead Array (CBA) technique according to the manufacturer’s protocols. In brief, 100μL of supernatant from each well was collected, centrifuged at 300×g for 5 min to remove cell debris, and stored at -80 °C until analysis. For cytokine detection, the cytokine detection kit (281601HN, Wellgrow, CA, USA) was employed. Capture beads specific to the target cytokines were mixed with the supernatant and incubated for 2 h at room temperature in the dark. Subsequently, PE-conjugated detection antibodies were added and incubated at room temperature for 1 h. Flow cytometry was performed to quantify cytokine concentrations based on standard curves using the corresponding cytometric bead array analysis software.

#### 2.9.4 Serum cytokine analysis in mice

Mouse serum samples were analyzed and compared across experimental groups using the RayPlex^®^ Mouse Cytotoxic T Cell Array Kit 1 (RayBiotech) according to the manufacturer’s kit protocols.

### 2.10 Establishment and treatment of NSCLC xenograft models

#### 2.10.1 A549 subcutaneous tumor model

All animal procedures were approved by the Animal Ethics Committee of Anhui Medical University (Approval No. LLSC20241619). Four-week-old female BALB/c nude mice (weight: 14–18 g) were obtained from SPF (Suzhou) Biotechnology Co., Ltd. After a one-week acclimatization period, A549 cells (8×10^6^ cells/mouse) were injected into the right axillary region of nude mice to establish subcutaneous xenograft tumors.

#### 2.10.2 Sample size, dosages, and timeline of drug administration

When the median tumor volume reached 100 mm³ (designated as day 0), the animals were randomly divided into five groups (n=5/group):

- Control (PBS)
- PD-1 antibody (Tislelizumab)
- PD-1 antibody + NK cells
- PD-1 antibody + Ad-anti-TGF-βRII
- PD-1 antibody + Ad-anti-TGF-βRII + NK cells

Treatments were administered as follows:

- Ad-anti-TGF-βRII: 5 × 10^7^ PFU/mouse, intratumoral (Days 0, 3, 6, 9)
- NK cells: 1 × 10^7^ cells/mouse, intravenous (Days 1, 2, 3, 9, 10, 11)
- PD-1 antibody: 0.2 mg/mouse Tislelizumab, intraperitoneal (Days -1, 2, 5, 8, 11)

To simulate the mechanical dissociation promoted by local virus injections, control animals were injected intratumorally with PBS on the same days as the virus treatments.

#### 2.10.3 Tumor monitoring and endpoint analysis

Tumor volume was measured every two days using digital calipers and the body weight was recorded at the same time. Tumor volume was calculated based on the formula: tumor volume = width^2^ × length/2. Relative tumor volume (RTV) was calculated by dividing the tumor volume on a given day by the volume on day 0 to evaluate the morphometric growth kinetics of the tumors. Antitumor activity was expressed as the tumor growth inhibition (TGI) percentage, which was calculated with the following formula: TGI (%) = [1 − (RTV of the treated group)/(RTV of the control group)] × 100 (%). On Day 12, mice were first deeply anesthetized by intraperitoneal injection of sodium pentobarbital (30 mg/kg), and then immediately euthanized by cervical dislocation. Then, tumors and organs were harvested for histopathology and RT-qPCR analysis. Serum was collected and stored at −80 °C.

### 2.11 Western blotting

After plasmid transfection or viral infection, 2.5×10^5^ cells from each group were cultured in serum-free medium for 3 h, followed by treatment with 100 ng/mL TGF-β1 (Chamot, Shanghai, China) for 30 min, with no TGF-β1 as controls. Parallelly, proteins were extracted from mouse tumor tissues. Both cellular and tissue samples were washed twice with ice-cold PBS and lysed in RIPA buffer (Solarbio, Beijing, China) supplemented with protease and phosphatase inhibitors according to the manufacturer’s specifications. Protein concentrations were quantified using a BCA assay kit (GLPBIO, CA, USA). Proteins were resolved by 10% SDS-PAGE (80 V for 30 min, then 120 V for 90 min) and electrotransferred to PVDF membranes (Immobilon^®^-P, IPVH00010; MilliporeSigma, MA, USA) at 350 mA for 80 min. Membranes were blocked with 5% bovine serum albumin (BSA) in TBST (Tris-buffered saline with 0.1% Tween-20) for 2 h at room temperature, followed by overnight incubation at 4 °C with primary antibodies diluted in TBST: phospho-SMAD2 (Affinity, Cat# AF3449), SMAD2/3 (Cell Signaling Technology, Cat# 3102S), and GAPDH (Proteintech, Cat# 10494-1-AP). After three 10-minute washes in TBST, membranes were probed with horseradish peroxidase (HRP)-conjugated goat anti-rabbit IgG (Proteintech, Cat# SA00001-2) for 1 h at room temperature and washed again as described above. Protein bands were visualized using MK Hypersensitive ECL Luminescent Solution (Biomiky, Shanghai, China). Image J software was used to quantify and analyze the relative band density.

### 2.12 Multimethod tissue analysis

#### 2.12.1 Histopathological examination

Tumor tissues and major organs (heart, liver, lung, kidney) from mice across treatment groups were fixed in 4% paraformaldehyde (Biosharp, Anhui, China) at 4 °C for 72 h. Subsequently, the tissues were processed for paraffin embedding and wax block sectioning, and then stained with hematoxylin and eosin (H&E) for histopathological assessment.

#### 2.12.2 Immunofluorescence

Tumor tissues were incubated with primary antibody (CD3, Servicebio, Cat# GB13014-50) for 2 h at room temperature, followed by Alexa Fluor 488 anti-rabbit secondary antibody (Abcam, ab150077) for 2 h. Tumor tissues were then stained with CD56 antibody (Servicebio, Cat# GB150119-50) for 2 h, followed by Alexa Fluor 594 anti-rabbit secondary antibody (Abcam, ab150080). Nuclei were counterstained with DAPI, and images were captured using a Leica SP5 confocal microscope. For quantitative analysis, three random high-power fields per section were selected. The number of CD3^-^CD56^+^ cells (representing NK cells) per field was counted using ImageJ software.

#### 2.12.3 Immunohistochemistry

Tumor tissues were incubated with primary antibodies against CD4 (Abcam, clone EPR6855, ab133616), CD8 (Abcam, clone EPR21769, ab217344), PD-1 (Abcam, clone NAT105, ab52587) and then with the HRP-conjugated secondary antibody goat anti-rabbit IgG H&L (Abcam, ab6721) to examine the distribution of CD4^+^ T cells and CD8^+^ T cells in the tumor tissues of mice from different treatment groups. Additionally, differences in PD-1 protein expression levels among tumor tissues were analyzed. Washing, DAB color development, hematoxylin counterstaining, and section mounting were then sequentially performed. Three high-power fields were randomly selected from each section for quantitative analysis of immunohistochemical results by ImageJ, and the percentage of positive staining area (area %) was calculated.

#### 2.12.4 Serum analysis for liver toxicity evaluation

At the endpoint, serum was harvested to measure lactate dehydrogenase (LDH) and alanine transaminase (ALT) levels, serving as *in vivo* indicators of potential toxicity.

### 2.13 RT-qPCR

Total RNA was extracted from cell samples and murine tissues using the RNAfast200 Total RNA Extraction Kit (Fastagen Biotechnology Co., Ltd., Shanghai, China) following the manufacturer’s protocol. Extracted RNA was then reverse-transcribed into cDNA with the PrimeScript™ RT Reagent Kit (with gDNA Eraser; Takara Bio, Kusatsu, Japan), which includes genomic DNA removal prior to reverse transcription. The 20 μL reverse transcription reaction system consisted of 10.0 μL of total RNA, 1.0 μL of PrimeScript RT Enzyme Mix I, 1.0 μL of RT Primer Mix, 4.0 μL of 5× PrimeScript Buffer 2 (for Real Time), and 4.0 μL of RNase-free water. Quantitative PCR was performed using the 2×Q3 SYBR qPCR Master Mix (Universal) and the CFX Connect Real-Time System (Bio-Rad, USA). Primer sequences for RT-qPCR are listed in **Table 1**. GAPDH was used as an internal reference and relative expression levels were calculated using the 2^−ΔΔCT^ method.

**Table 1:** Primer sequences for qPCR.

Gene	Sequences (5′ to 3′)
anti-TGF-βRII (Human)	Forward: ATGAGGCTCCCTGCTCAGCT
	Reverse: AGATGGTGCAGCCACAGTTC
Perforin (Mouse)	Forward: CTGCCACTCGGTCAGAATG
	Reverse: CGGAGGGTAGTCACATCCAT
GZMB (Mouse)	Forward: TCTCGACCCTACATGGCCTTA
	Forward: TCCTGTTCTTTGATGTTGTGGG
IFN-γ (Mouse)	Forward: GCCACGGCACAGTCATTGA
	Reverse: TGCTGATGGCCTGATTGTCTT
HIF-1α (Mouse)	Forward: TCTCGGCGAAGCAAAGAGTC
	Reverse: AGCCATCTAGGGCTTTCAGATAA
GAPDH (Human)	Forward: GAAGGTGAAGGTCGGAGTC
	Reverse: GAAGATGGTGATGGGATTTC

### 2.14 ELISA

NK cell culture supernatants were collected after centrifugation (500×g, 5 min). For the BALB/c nude mice tumor-bearing animal model, terminal blood was collected under isoflurane anesthesia via orbital enucleation. After 1 h incubation at room temperature, samples were centrifuged at 4 °C for 20 min. Perforin concentrations in serum and culture supernatants were measured using Human and Mouse PRF1(Perforin 1) ELISA Kit (Elabscience, Wuhan, China) according to the manufacturer’s protocol. Absorbances at 450 nm were detected with the microplate reader (Tecan, Männedorf, Switzerland).

### 2.15 Statistical analysis

All *in vitro* experiments were independently repeated at least three times unless otherwise specified. For *in vivo* studies, experimental group comparisons and animal cohort sizes are detailed in the corresponding figure legends. Statistical analyses were performed using GraphPad Prism v9.0 (GraphPad Software, San Diego, CA, USA). Inter-group differences were assessed by one-way ANOVA or two-way ANOVA with Tukey’s multiple comparison test. A significance level of *P* < 0.05 was considered significant.

## 3 Results

### 3.1 Generation of oncolytic adenoviruses encoding TGF-β inhibitor

**Figure 1:**
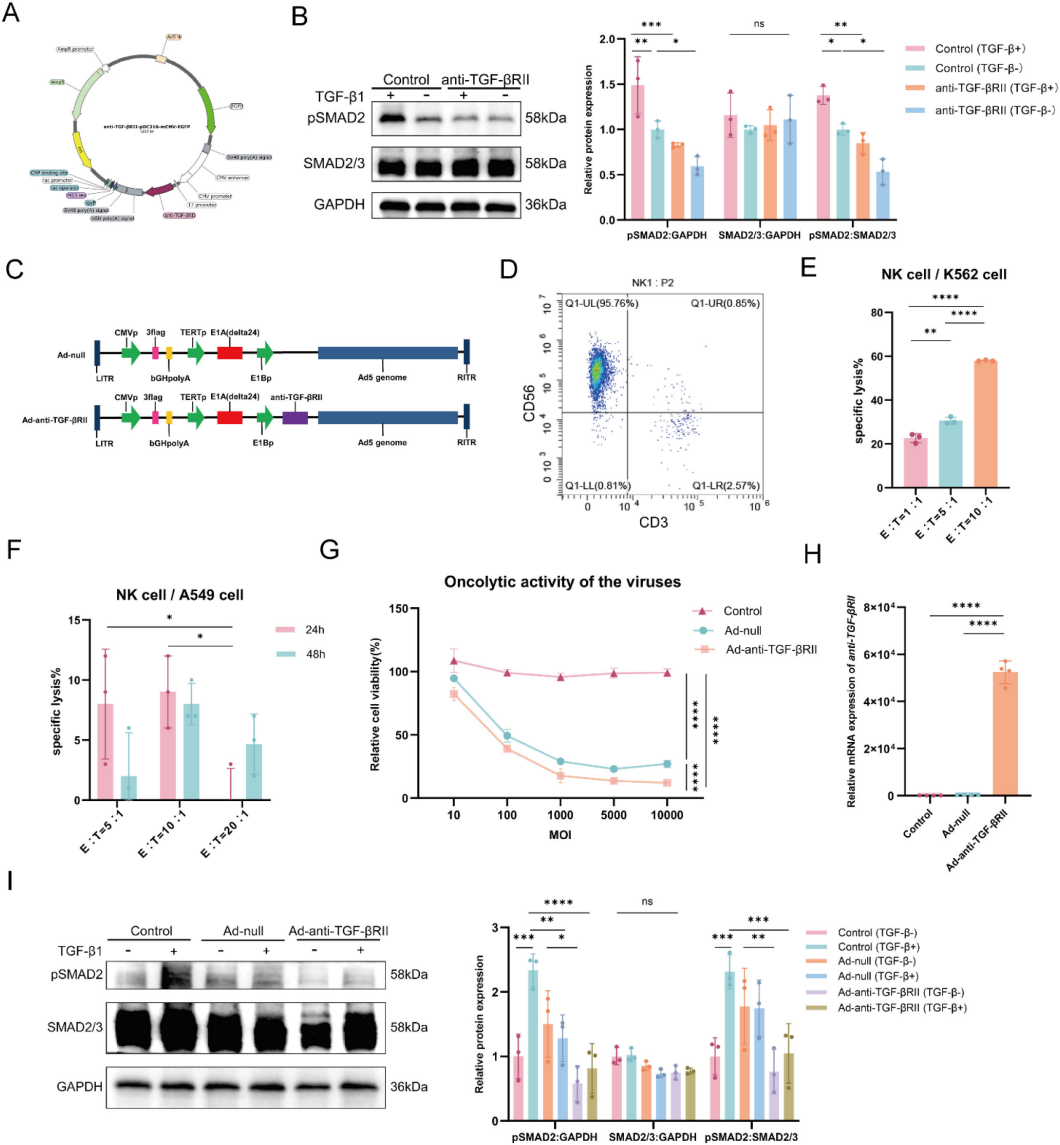
Generation of key components and validation of the core anti-tumor mechanism. **(A)** Plasmid map for overexpression of anti-TGF-βRII gene. **(B)** Immunoblot and densitometry of pSMAD2 signaling, downstream of TGF-β, in A549 cells, treated with recombinant TGF-β1 and A549 cells infected with plasmid-anti-TGF-βRII, or mock-infected. **(C)** The scheme of Ad-null (control) and Ad-anti-TGF-βRII. **(D)** The percentage of *in vitro*-cultured NK cells (CD3^−^CD56^+^) on day 14 was detected by flow cytometry. The cytotoxic activity of NK cells was assessed against K562 cells (24 h co-culture) **(E)** and A549 cells **(F)** target cells at various effector-to-target (E:T) ratios. **(G)** Dose-dependent oncolytic activity of Ad-null and Ad-anti-TGF-βRII in A549 cells 48 h post-infection. **(H)** RT-qPCR analysis confirming specific upregulation of anti-TGF-βRII mRNA in A549 cells infected with Ad-anti-TGF-βRII, but not Ad-null. **(I)** Western blot analysis demonstrating that Ad-anti-TGF-βRII infection, but not Ad-null, significantly reduces pSMAD2 levels in A549 cells. *p < 0.05; **p < 0.01; ***p < 0.001; ****p < 0.0001; ns means no significance by one-way ANOVA or two-way ANOVA with Tukey’s multiple comparison test. Error bars indicate SDs (n=3).

A plasmid designed to overexpress a TGF-β inhibitor was constructed (**Figure 1A**). To validate its functionality, the plasmid was transfected into A549 cells (**Supplementary Figure 2**). The analysis confirmed that TGF-β signaling was reduced in transfected A549 cells, both with and without exogenous TGF-β stimulation, as shown by a significant decrease in pSMAD2 relative to total SMAD2/3, while total SMAD2/3 levels showed no significant difference (**Figure 1B**).

The oncolytic adenoviruses, Ad-null (control) and Ad-anti-TGF-βRII (**Figure 1C**), were subsequently generated after the construction of cloning vectors (**Supplementary Figure 3**) and viral packaging. The packaged viruses achieved high titers of 1 × 10¹¹ PFU/mL (**Supplementary Figure 4**).

### 3.2 Activation and expansion of peripheral blood-derived NK cells using a feeder cell system

Expanded NK cells exhibited a rapid growth phase by day 10 (**Supplementary Figure 5A**), culminating in a proliferation index of 350 after 14 days. Flow cytometry analysis demonstrated high purity, with >95% of cells expressing the NK cell phenotype (CD3^-^CD56^+^) (**Figure 1D**). To assess the cytotoxic potential of the expanded NK cells, we evaluated their activity against both K562 (leukemia) and A549 (lung adenocarcinoma) cell lines. Against K562 cells in a 24 h co-culture assay, NK cells exhibited peak cytotoxic activity at an effector-to-target (E:T) ratio of 10:1, achieving approximately 60% target cell lysis (**Figure 1E**). For A549 cells, peak cytotoxicity also occurred at an E:T ratio of 10:1 following a 24 h co-culture (**Figure 1F**). (Additional morphological characterization of the expanded NK cells is provided in **Supplementary Figure 5**).

### 3.3 Ad-anti-TGF-βRII demonstrates cytotoxic effects and modulates TGF-β signaling

Both oncolytic adenoviruses, Ad-null and Ad-anti-TGF-βRII, exhibited dose-dependent cytotoxicity against A549 lung adenocarcinoma cells (**Supplementary Figure 6**), with Ad-anti-TGF-βRII showing significantly enhanced oncolytic activity compared to Ad-null at equivalent MOIs (**Figure 1G**). Notably, at an MOI of 100, Ad-anti-TGF-βRII reduced A549 cell viability to 50% at 48 h.

To investigate whether the superior cytotoxicity of Ad-anti-TGF-βRII was mediated through TGF-β pathway modulation, we analyzed anti-TGF-βRII gene expression and downstream signaling. RT-qPCR revealed a strong upregulation of anti-TGF-βRII mRNA in Ad-anti-TGF-βRII-infected cells, while minimal expression was detected in Ad-null-treated or untreated controls (**Figure 1H**). Consequently, western blot analysis showed a significant downregulation of the key signaling mediator pSMAD2 (**Figure 1I**).

These findings demonstrate that Ad-anti-TGF-βRII exerts dual anti-tumor effects: direct oncolytic activity combined with specific inhibition of the TGF-β/SMAD pathway through anti-TGF-βRII overexpression. This combined mechanism leads to enhanced tumor cell cytotoxicity and growth suppression.

### 3.4 Ad-anti-TGF-βRII potentiates NK cell cytotoxicity and synergizes with PD-1 blockade *in vitro*

**Figure 2:**
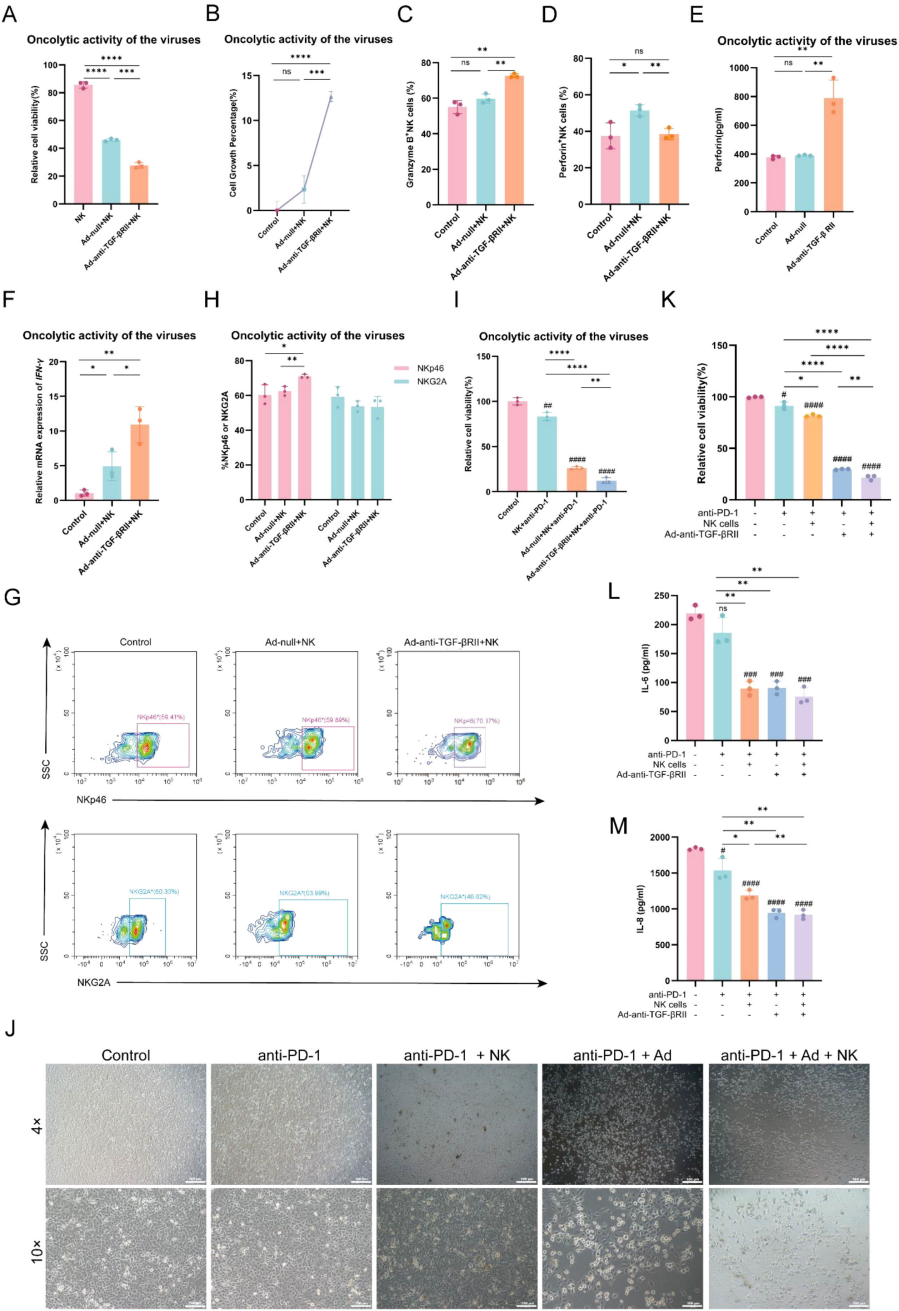
Ad-anti-TGF-βRII potentiates NK cell function and synergizes with PD-1 blockade. **(A)** Enhanced killing of A549 cells by NK cells following pre-infection of target cells with Ad-anti-TGF-βRII compared to Ad-null. **(B)** NK cells were treated with the supernatant of control or virus-infected A549 cells for 48 h, the growth rate of NK cells were determined. Flow cytometry detection of GZMB **(C)** and perforin **(D)** in NK cells. **(E)** Co-incubated supernatants were collected and assayed for perforin using the ELISA kit. **(F)** Collected NK cells and the expression of IFN-γ was evaluated by RT-qPCR assay. NK cells were pre-stained with NKG2A and NKp46, flow cytometry **(G)** was utilized to analyze the levels of receptor protein **(H, I)** Experimental design to evaluate triple-combination therapy, comparing Ad-null versus Ad-anti-TGF-βRII (100 MOI) as the viral pretreatment component. **(J)** After co-culture for 48 h, representative microscopic images of A549 cells after co-culture (4×: Scale bar=200 μm and 10×: Scale bar=100 μm). **(K)** The cell viability of A549 cells in each group was detected by CCK-8 assay. Meanwhile, the cell culture supernatant of each group was collected, and the secretion levels of IL-6 **(L)** and IL-8 **(M)** were quantitatively analyzed by flow cytometry. *p < 0.05; **p < 0.01; ***p < 0.001; ****p < 0.0001; ns means no significance by one-way ANOVA or two-way ANOVA with Tukey’s multiple comparison test. Error bars indicate SDs (n=3).

When A549 cells were pre-infected with either Ad-null or Ad-anti-TGF-βRII (MOI 100, 24 h) and subsequently co-cultured with NK cells (E:T ratio 10:1, 24 h), significantly enhanced tumor cell killing was observed, with Ad-anti-TGF-βRII demonstrating superior enhancement of NK cell-mediated cytotoxicity compared to Ad-null (**Figure 2A**). Conditioned medium from Ad-anti-TGF-βRII-infected (MOI 100, 48 h) A549 cells also potently stimulated NK cell proliferation during a subsequent 48 h culture (**Figure 2B**) and altered their activation profile. These preconditioned NK cells exhibited increased intracellular granzyme B, alongside a marked reduction in intracellular perforin that corresponded with a significant rise in perforin secretion into the supernatant (**Figures 2C–E**), indicative of active degranulation (30, 31). Mechanistically, the intracellular granzyme B accumulation reflects both retention due to incomplete perforin pore formation during degranulation and increased *de novo* synthesis following NK cell activation. Additionally, they showed elevated IFN-γ mRNA levels (**Figure 2F**). We also examined the expression of the inhibitory receptor NKG2A and activating receptor NKp46 (32). Flow cytometry analysis demonstrated marked upregulation of NKp46 in anti-TGF-βRII-treated NK cells relative to Ad-null controls (**Figures 2G, H**), while NKG2A expression remained unchanged. Finally, this optimized approach demonstrated synergy with PD-1 blockade. The combination of Ad-anti-TGF-βRII pre-infection, NK cell co-culture, and PD-1 antibody treatment achieved superior tumor cell killing compared to all other conditions (**Figure 2I**).

Collectively, Ad-anti-TGF-βRII enhances NK cell anti-tumor activity by promoting their proliferation, upregulating NKp46, increasing granzyme B, perforin and IFN-γ production, and synergizing with PD-1 blockade.

### 3.5 PD-1 antibody combined with NK cells and Ad-anti-TGF-βRII exhibits stronger anti-tumor effects *in vitro*

The combination of PD-1 blockade, NK cell therapy, and oncolytic virotherapy demonstrated potent anti-tumor effects against A549 cells. While PD-1 antibody monotherapy showed limited efficacy, combination strategies significantly enhanced cytotoxic effects. Both dual-combination groups (PD-1 antibody + NK cells or PD-1 antibody + Ad-anti-TGF-βRII) demonstrated superior tumor suppression compared to single-agent treatment. Most notably, the triple-combination (PD-1 antibody + NK cells + Ad-anti-TGF-βRII) exhibited the most potent anti-tumor activity, as confirmed by microscopic examination demonstrating enhanced tumor cell killing (**Figures 2J, K**). We further characterized treatment-induced changes in the tumor microenvironment by analyzing key inflammatory cytokines. Measurement of IL-6 and IL-8 (33, 34) levels revealed a significant reduction in all combination therapy groups (**Figures 2L, M**). Although cytokine suppression in the triple-combination group was comparable to that of the PD-1 antibody + Ad-anti-TGF-βRII dual therapy, the triple-combination approach was still considered as the most effective anti-tumor strategy *in vitro* due to its superior direct tumoricidal activity.

### 3.6 Triple-combination therapy with PD-1 blockade, NK cells, and TGF-βRII-targeted oncolytic adenovirus suppresses A549 xenograft growth

**Figure 3:**
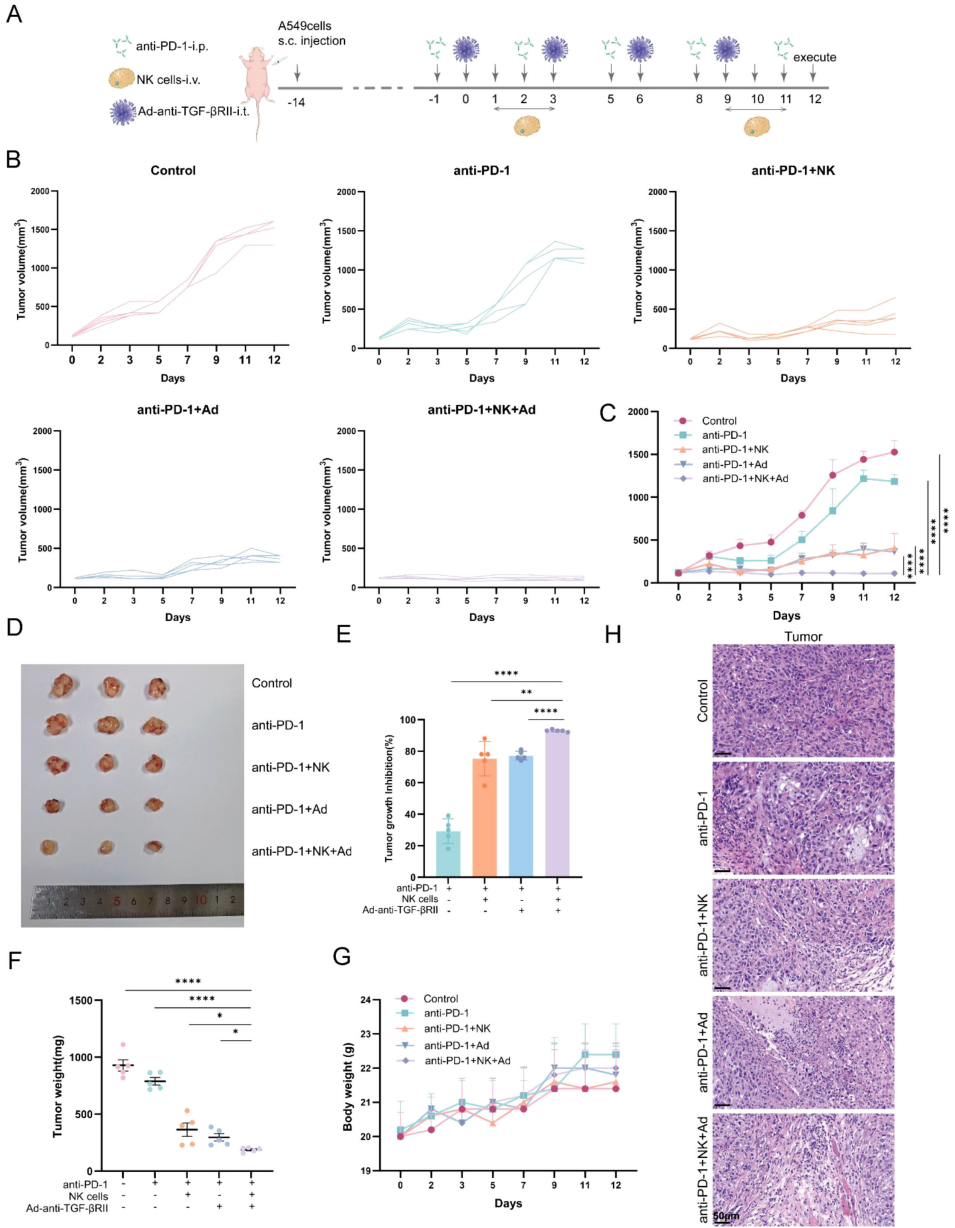
The triple-combination therapy with PD-1 antibody, NK cells, and Ad-anti-TGF-βRII evoked stronger antitumor responses than other treatments (n=5). To establish A549 subcutaneous tumor model, 8×10^6^ A549 cells were injected subcutaneously into BALB/c mice. Fourteen days after the cell injection, tumor-bearing mice were divided into 5 groups without statistical difference in tumor volume (n=5). Virus intratumoral injections (5×10^7^ PFU/mouse) with Ad-anti-TGF-βRII were given on experiment days 0, 3, 6, and 9. NK cells (1×10^7^ cells/mouse) via tail vein injection on days 1, 2, 3, 9, 10, and 11. PD-1 antibody (Tislelizumab, 0.2 mg/mouse) via intraperitoneal on days -1, 2, 5, 8, and 11. Experiment was finished on day 12. **(A)** Experimental timeline for *in vivo* studies. Tumor volumes were monitored every two days and are presented in **(B)** and **(C)** (n=5). **(D)** Representative tumor images (n=3) at the experiment endpoint (day 15). **(E)** Tumor inhibition rate at the experimental endpoint (n=5). **(F)** Tumor weight at the experimental endpoint (n=5). **(G)** The body weight of mice was monitored every two days (n=5). **(H)** The histopathological changes in tumor tissues were analyzed by H&E staining. Scale bar=50 μm. *p < 0.05; **p < 0.01; ****p < 0.0001 by one-way ANOVA or two-way ANOVA with Tukey’s multiple comparison test. Error bars indicate SEMs (n=5).

The combined therapeutic strategy of PD-1 blockade, NK cell immunotherapy and TGF-βRII-targeted oncolytic virotherapy demonstrated significant antitumor efficacy in A549 xenograft models. When treatments began on tumors of approximately 100 mm³ (**Figure 3A**), the PD-1 antibody monotherapy only inhibited tumor growth by 28.83%. In contrast, the combination therapies were substantially more effective: adding NK cells achieved 74.98% inhibition, while adding Ad-anti-TGF-βRII achieved 77.32% inhibition. Notably, the triple combination therapy (PD-1 antibody + NK cells + Ad-anti-TGF-βRII) exhibited superior tumor suppression with 93.09% growth inhibition (*P* < 0.01 versus all other groups; **Figures 3B–E**), corresponding to a 79.78% reduction in final tumor weight compared to controls (187.6 mg versus 927.6 mg; **Figure 3F**). All treatment regimens were well-tolerated, with no significant body weight changes observed (**Figure 3G**). Histopathological analysis confirmed that the triple combination induced pronounced inflammatory infiltration and extensive tumor necrosis (**Figure 3H**). These findings highlight the synergistic potential of combining immune checkpoint inhibition with cellular immunotherapy and targeted virotherapy for NSCLC treatment.

### 3.7 Ad-anti-TGF-βRII reprograms the tumor immune microenvironment to augment NK cell cytotoxicity and lymphocyte infiltration

**Figure 4:**
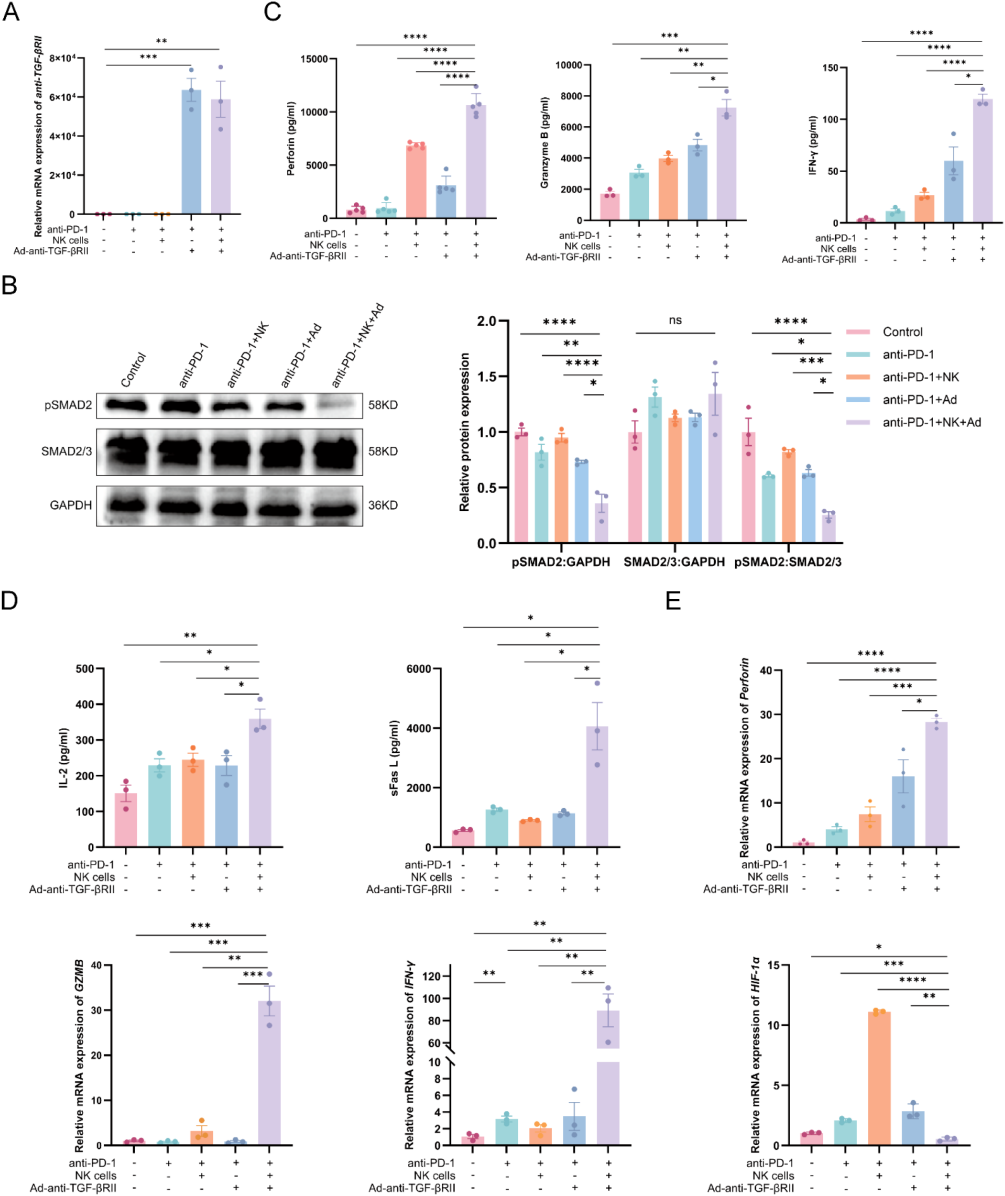
Detection of key molecules in antitumor immune response. **(A)** RT**-**qPCR detection of anti-TGF-βRII expression in tumor tissues. **(B)** Western blotting detection of the expression level changes of pSMAD2 in each group. **(C)** Detection of the concentration of perforin, GZMB, IFN-γ in mouse serum at the end of the experiment. **(D)** Concentration of sFas L, IL-2 in mouse serum. **(E)** RT**-**qPCR detection of perforin, GZMB, IFN-γ, HIF-1α expression in tumor tissues. *p < 0.05; **p < 0.01; ***p < 0.001; ****p < 0.0001; ns means no significance by one-way ANOVA with Sidak’s multiple comparison test. Error bars indicate SEMs (n=3).

Analysis of tumor tissues confirmed the *in vivo* activity of the therapeutic transgene and its target. Anti-TGF-βRII mRNA was detected exclusively in virus-treated groups, and correspondingly, TGF-β/SMAD signaling (pSMAD2) was most strongly suppressed in the triple-combination group (**Figures 4A, B**). Collectively, these data confirm successful *in vivo* transgene delivery, significant suppression of pSMAD2 in treated tumors, and enhanced TGF-β pathway blockade with combination therapy, aligning with our *in vitro* observations.

The triple-combination therapy demonstrated superior immunostimulatory effects compared to all other treatment groups. Serum analysis revealed significantly elevated levels of cytotoxic mediators (perforin:10376.9 ± 346.9 pg/mL; GZMB: 7244.6 ± 747.9 pg/mL and IFN-γ: 119.6 ± 6.6 pg/mL) (all *P* < 0.01 vs monotherapy; **Figure 4C**). Corresponding increases were observed in tumor microenvironment cytokines, with sFas L (4063.8 ± 1124.3 pg/mL) and IL-2 (359.0 ± 39.0 pg/mL) showing the highest concentrations in the triple therapy group (**Figure 4D**). Transcriptional profiling of tumor tissues revealed marked upregulation of perforin (28.3 ± 1.2-fold, *P* < 0.0001), GZMB (32.1 ± 5.7-fold, *P* < 0.001), and IFN-γ (89.3 ± 20.9-fold, *P* < 0.01), alongside a reduction in HIF-1α (47.6% ± 8.3%, *P* < 0.05) (**Figure 4E**). These molecular changes indicate enhanced antitumor immune responses coupled with improved tumor microenvironment conditions.

**Figure 5:**
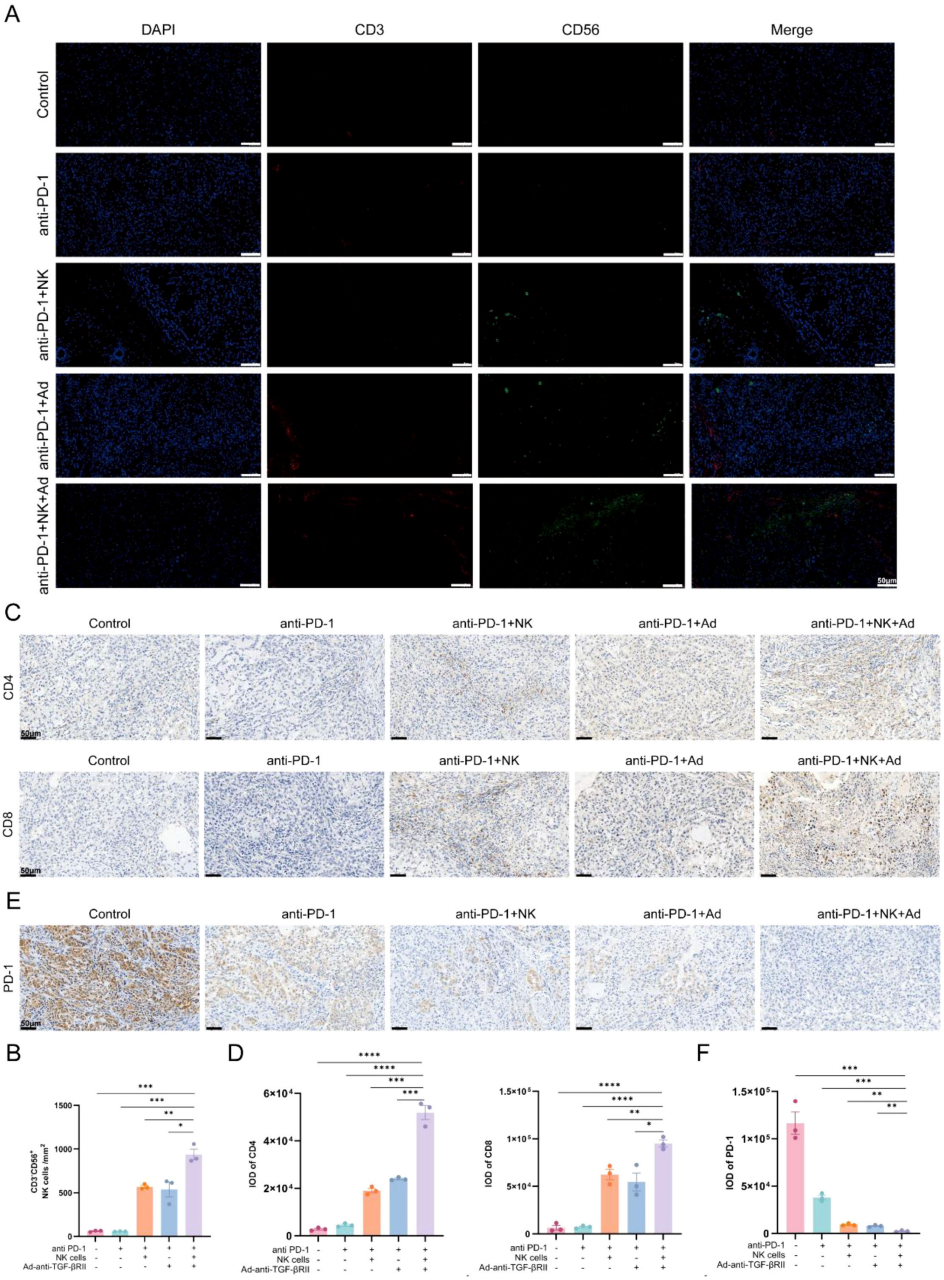
Multimethod detection and quantitative analysis of immune cell subsets within tumor tissues. **(A)** Immunofluorescence (IF) detection of NK cells (CD3^-^CD56^+^) in tumor tissues per field. Scale bar = 50 µm. **(B)** Statistical analysis of the number of CD3^-^CD56^+^ cells. Immunohistochemistry (IHC) staining for CD4^+^, CD8^+^ T cells **(C)** and PD-1 **(E)** in tumor tissues. Scale bar = 50 µm. Statistical results of the positive area ratio for CD4^+^, CD8^+^ T cells **(D)** and PD-1 **(F)**. *p < 0.05; **p < 0.01; ***p < 0.001; ****p < 0.0001 by one-way ANOVA with Sidak’s multiple comparison test. Error bars indicate SEMs (n=3).

Analysis of immune cell infiltration revealed distinct patterns. Immunofluorescence (IF) staining for CD3^-^CD56^+^ cells confirmed the specific recruitment of NK cells into tumor tissues receiving combination therapies, with the most intense signal in the triple-combination group (**Figures 5A, B**). Similarly, immunohistochemistry (IHC) showed that infiltration of CD4^+^ and CD8^+^ T cells also peaked in this group (**Figures 5C, D**), while PD-1 expression was substantially diminished (**Figures 5E, F**).

These results demonstrate that the triple combination therapy orchestrates a coordinated immune response through enhanced NK cell recruitment and cytotoxicity, promoted effector T cell infiltration, and reduced PD-1-mediated immunosuppression.

### 3.8 Combination therapy with PD-1 antibody, NK cells and Ad-anti-TGF-βRII demonstrates favorable safety profile

**Figure 6:**
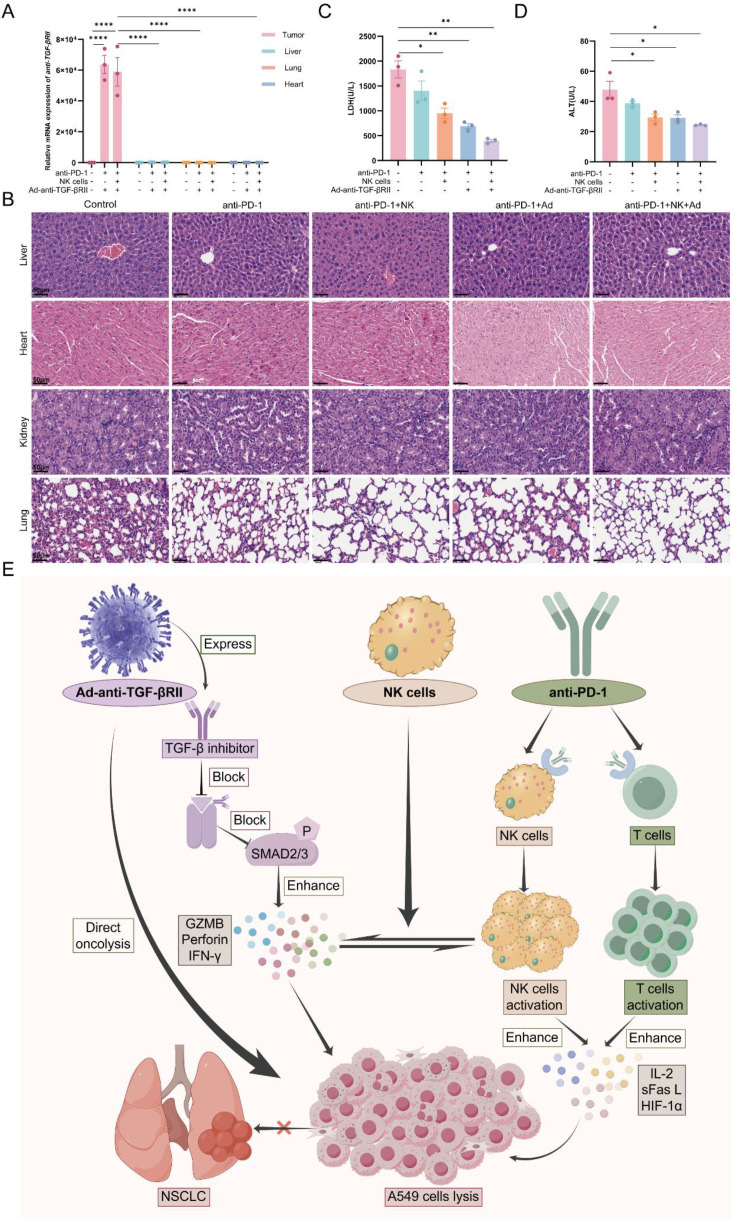
Combined therapy with PD-1 antibody, NK cells, and Ad-anti-TGF-βRII does not increase the toxicity in xenograft model. **(A)** RT-qPCR detection of anti-TGF-βRII expression in tumor, liver, lung, and heart. **(B)** Representative H&E-stained images of the liver, heart, kidney, and lung. Scale bar=50 μm. **(C)** Serum LDH and ALT **(D)** results of mice at the end of the experiment. **(E)** Mechanism of synergistic anti-tumor activity from a triple-combination therapy. Created in FigDraw (www.figdraw.com). *p < 0.05; **p < 0.01; ***p < 0.001; ****p < 0.0001 by one-way ANOVA with Sidak’s multiple comparison test (a, c and d). Error bars indicate SEMs (n=3).

To evaluate the safety profile of the combination immunotherapy (PD-1 antibody + NK cells + Ad-anti-TGF-βRII), we first assessed the tissue specificity of the oncolytic adenovirus. Using the virally encoded anti-TGF-βRII transgene as a molecular tracer, RT-qPCR analysis demonstrated restricted viral replication to tumor tissues, with no detectable anti-TGF-βRII mRNA in normal organs including liver, lungs, or heart (**Figure 6A**). These results confirm the tumor-selective tropism of Ad-anti-TGF-βRII and its inability to disseminate systemically.

Histopathological analysis of major organs (liver, heart, kidneys, and lungs) showed preserved tissue architecture with no evidence of treatment-related abnormalities following the combination therapy (**Figure 6B**). The absence of pathological changes in these critical organs indicates a favorable safety profile for this multimodal regimen, which includes intratumoral viral administration, intravenous NK cell delivery, and systemic PD-1 antibody treatment. Correspondingly, serum levels of injury markers (LDH and ALT) were significantly reduced in the treatment group (**Figures 6C, D**). These findings, together with the normal hepatic architecture observed in H&E-stained sections (**Figure 6B**), demonstrate the absence of treatment-induced hepatotoxicity.

To synthesize these findings into a unified model, we propose a mechanistic framework for the observed synergy (**Figure 6E**). Our data demonstrate that, in addition to its direct oncolytic effect, intratumoral administration of Ad-anti-TGF-βRII remodels the TME by blocking TGF-β signaling, which in turn primes adoptively transferred NK cells and enhances their cytotoxic function. This effect is further amplified by systemic PD-1 blockade, which synergistically overcomes ICI resistance while maintaining a favorable safety and efficacy profile.

## 4 Discussion

The core finding of this study is that a novel triple-combination therapy, comprising a PD-1 antibody, adoptive Natural Killer (NK) cells, and an oncolytic adenovirus armed with a TGF-β inhibitor (Ad-anti-TGF-βRII), generates significant synergistic anti-tumor effects in models of non-small cell lung cancer (NSCLC). We have demonstrated that this synergy is driven by a comprehensive remodeling of the tumor microenvironment (TME), transforming it from an immunosuppressive to an inflamed state. This discovery offers a promising new strategy to overcome the profound challenge of resistance to immune checkpoint inhibitors (ICIs), a major obstacle in the treatment of recurrent and metastatic NSCLC (35, 36).

The strategy was built upon validating each component’s contribution to the final synergistic outcome. A foundational element of this therapy was the engineered oncolytic adenovirus itself. Our results first confirmed the potent, dose-dependent oncolytic activity of Ad-anti-TGF-βRII against NSCLC cells, establishing its direct tumoricidal capacity. More importantly, we validated its intended immunomodulatory function. The virus-expressed transgene was shown to effectively inhibit the TGF-β/SMAD signaling pathway, evidenced by a significant reduction in phosphorylated SMAD2. This is a critical finding, as TGF-β signaling pathway is involved in cancer immune escape and ICI resistance, and TGF-β significantly inhibits NK cell function by blocking IFN-γ expression (37). These initial results established Ad-anti-TGF-βRII as a functional, dual-mechanism agent capable of both direct tumor lysis and targeted immunomodulation.

Having validated the virus, we next sought to interpret its direct impact on NK cells, addressing a major barrier to adoptive cell therapy in solid tumors. While our expanded NK cells demonstrated high purity and potent baseline cytotoxicity, their function is known to be blunted by the potent immunosuppressive properties of TME (38). Our findings clearly show that Ad-anti-TGF-βRII directly counteracts this suppression. The enhancement of NK cell proliferation, upregulation of the activating receptor NKp46, and increased production and secretion of perforin and GZMB following exposure to the virus-conditioned microenvironment provide a mechanistic explanation for the therapeutic synergy (39–41). These results demonstrate that the virus-mediated blockade of local TGF-β signaling translates directly into a functional revitalization of NK cell anti-tumor activity, effectively priming them for a more potent attack and overcoming a key limitation of their standalone use (42–45).

Building on the enhanced functionality of these “revitalized” NK cells, we next evaluated the full triple-combination therapy. The *in vitro* data provided the first clear indication of synergy, where the combination of the PD-1 antibody, NK cells, and Ad-anti-TGF-βRII demonstrated the most potent tumor-killing activity, significantly outperforming any dual-combination. This suggests a multi-layered attack: the oncolytic virus weakens the tumor’s defenses by inhibiting TGF-β, which in turn unleashes the cytotoxic potential of the NK cells, whose efficacy is further sustained by the blockade of the PD-1 checkpoint.

The full therapeutic potential of this strategy was most powerfully realized in our *in vivo* xenograft model, where the triple-combination therapy achieved a striking 93.09% tumor growth inhibition, a result far superior to any other treatment group. We attribute this potent synergy to the profound and multifaceted TME remodeling orchestrated by the oncolytic virus. Consistent with a growing body of literature, oncolytic viruses can act as *in situ* vaccines, initiating a cascade of inflammatory signals that can transform immunologically “cold” non-responsive tumors into “hot” immune-infiltrated tumors (46). Our data provide compelling evidence for this phenomenon. The triple therapy led to the most significant increases in both systemic and intratumoral levels of cytotoxic effector molecules. Most importantly, beyond stimulating dendritic cell (DC) maturation, local inflammatory changes induced by oncolytic virus infection promote DC migration to the spleen and draining lymph nodes. This facilitates the presentation of tumor-associated antigens to T cells, eliciting infiltration of antitumor CD4^+^ and CD8^+^ T cells that mediate tumor-specific cytotoxicity (47–49). Critically, these tumor-infiltrating T cells are derived from the host’s endogenous pool, activated *in situ* rather than being adoptively transferred, which aligns with the high purity (>95%) of our infused NK cell product. This transition of the tumor landscape into a site of active inflammation, populated by both innate and adaptive effector lymphocytes, is the likely mechanistic underpinning for overcoming ICI resistance. It creates a favorable environment where the PD-1 antibody can function more effectively, not just on NK cells but also on the newly recruited T cells.

From a clinical and translational standpoint, a key finding of this study is the favorable safety profile of this complex, multi-modal regimen. A significant concern for any therapy involving a replicating virus is the risk of off-target effects. Our analysis confirmed the robust tumor-selective tropism of Ad-anti-TGF-βRII, with its therapeutic transgene expression being restricted to the tumor tissue and undetectable in major organs. Furthermore, comprehensive histopathological and serum biochemical analyses revealed no evidence of treatment-related organ damage or systemic toxicity. For a sophisticated therapy that combines three distinct and powerful biological agents—a virus, an allogeneic cell population, and a monoclonal antibody—demonstrating such a high degree of safety and tolerability is a crucial step and provides a strong foundation for its potential translation into the clinic (50).

Nevertheless, several limitations of this study should be noted. First, the use of immunodeficient BALB/c nude mice prevented a full assessment of immune interactions, including adaptive responses and the balance between antiviral and antitumor immunity (51, 52). Second, immune cell analysis was confined to histological methods without the complementary use of flow cytometry, thereby limiting the precision of quantification and the depth of immunophenotyping. Future work should employ immunocompetent syngeneic models with fresh-tissue sampling to better elucidate immune dynamics, synergy mechanisms, and optimal treatment regimens.

## 5 Conclusions

This study presents a novel and potent triple-combination immunotherapy for NSCLC. Our data provide a clear mechanistic rationale for its efficacy, demonstrating that the strategy works through multiple, synergistic modes of action. The oncolytic virus not only directly kills tumor cells but also enhances NK cell function by inhibiting TGF-β, and together they remodel the immunosuppressive TME into an inflamed, “hot” environment conducive to a powerful, multi-pronged immune attack. These findings offer a highly promising new strategy for treating patients with advanced NSCLC, particularly those resistant to existing ICI therapies, and provide a strong rationale for future clinical trials.

## Data Availability

The raw data supporting the conclusions of this article will be made available by the authors, without undue reservation.

## Supplementary material

Supplementary Figure 1 A549 cells were treated with different concentrations of PD-1 antibody for 24h or 48h, and the cell viability of A549 cells was detected by CCK-8 method. ****p < 0.0001 by one-way ANOVA with Tukey’s multiple comparison test; ns means no significance. Error bars indicate SDs (n=3).

Supplementary Figure 2 Transfect the plasmid carrying EGFP fluorescence into A549 cells and observe the fluorescence under a fluorescence microscope (4×: Scale bar=200 μm and 10×: Scale bar=100 μm).

Supplementary Figure 3 Construction of overexpression adenovirus vectors.

Supplementary Figure 4 End-point dilution method to determine the titer of the virus (4×: Scale bar=200 μm).

Supplementary Figure 5 The expansion index and morphological characteristics of natural killer (NK) cells. **(A)** Growth curve of NK cells. **(B)** Observation of NK cell morphology under a 4× objective lens of an inverted microscope on day 7 and day 10 of *in vitro* expansion (4×: Scale bar=200 μm). **(C)** Morphology of NK cells under an inverted microscope on day 14 of *in vitro* expansion (4×: Scale bar=200 μm and 10×: Scale bar=100 μm).

Supplementary Figure 6 A549 cells were infected with Ad-null and Ad-anti-TGF-βRII at MOI of 10, 10, 1000, 10000 for 48 h and then photographed. Pictures were taken 48 h later. Dead or dying cells could be identified by their rounded (4×: Scale bar=200 μm).
