# Supplementary figures and images for: Oncolytic adenovirus encoding a TGF-β inhibitor synergizes with PD-1 blockade to potentiate NK cell cytotoxicity against NSCLC
Source: Front Immunol. 2026 Feb 25;17:1759236. doi: 10.3389/fimmu.2026.1759236 (PMC12975894; doi:10.3389/fimmu.2026.1759236)

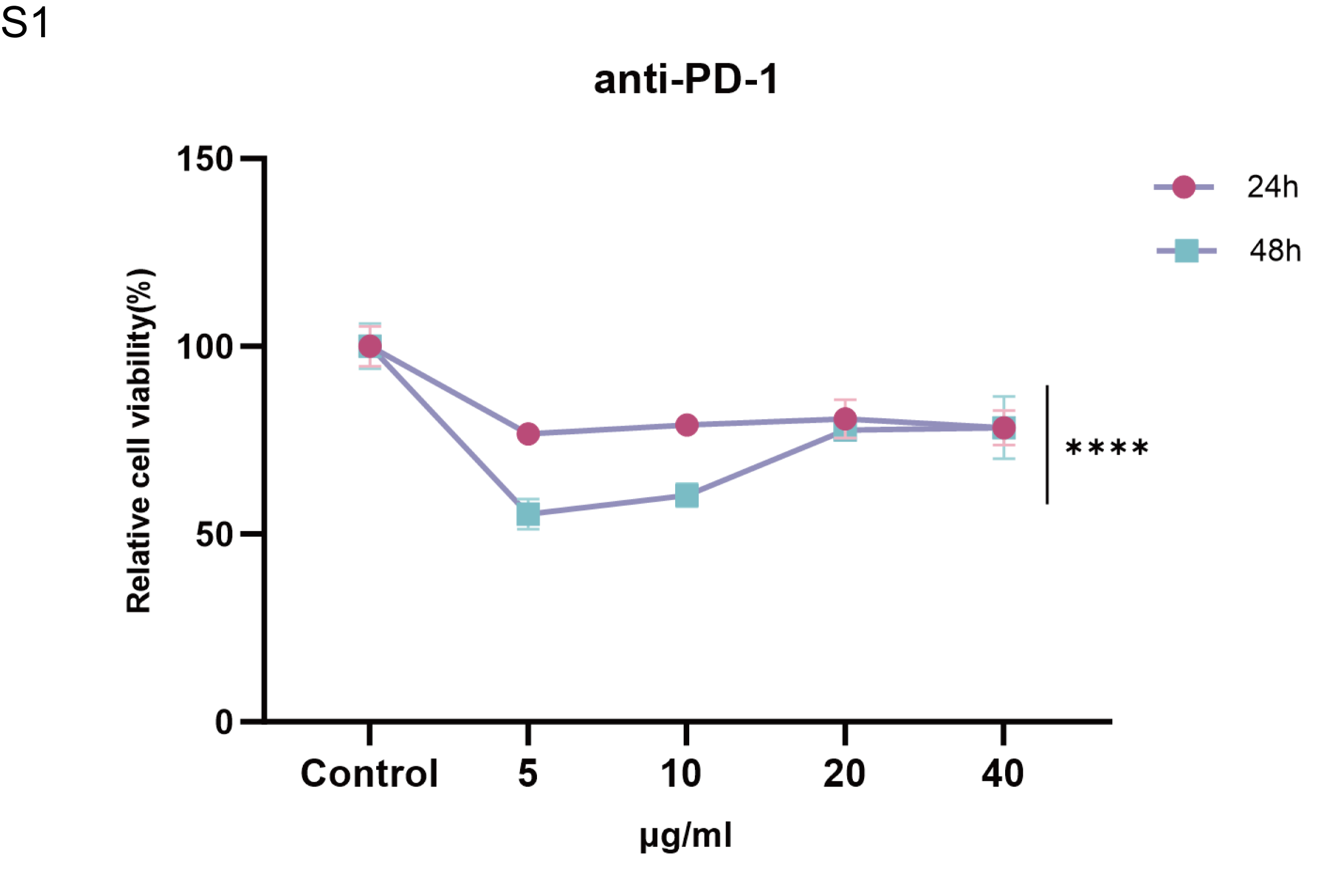

Supplement: Supplementary Figure 1 — A549 cells were treated with different concentrations of PD-1 antibody for 24h or 48h, and the cell viability of A549 cells was detected by CCK-8 method. ****p < 0.0001 by one-way ANOVA with Tukey’s multiple comparison test; ns means no significance. Error bars indicate SDs (n=3). [file Image1.tif]

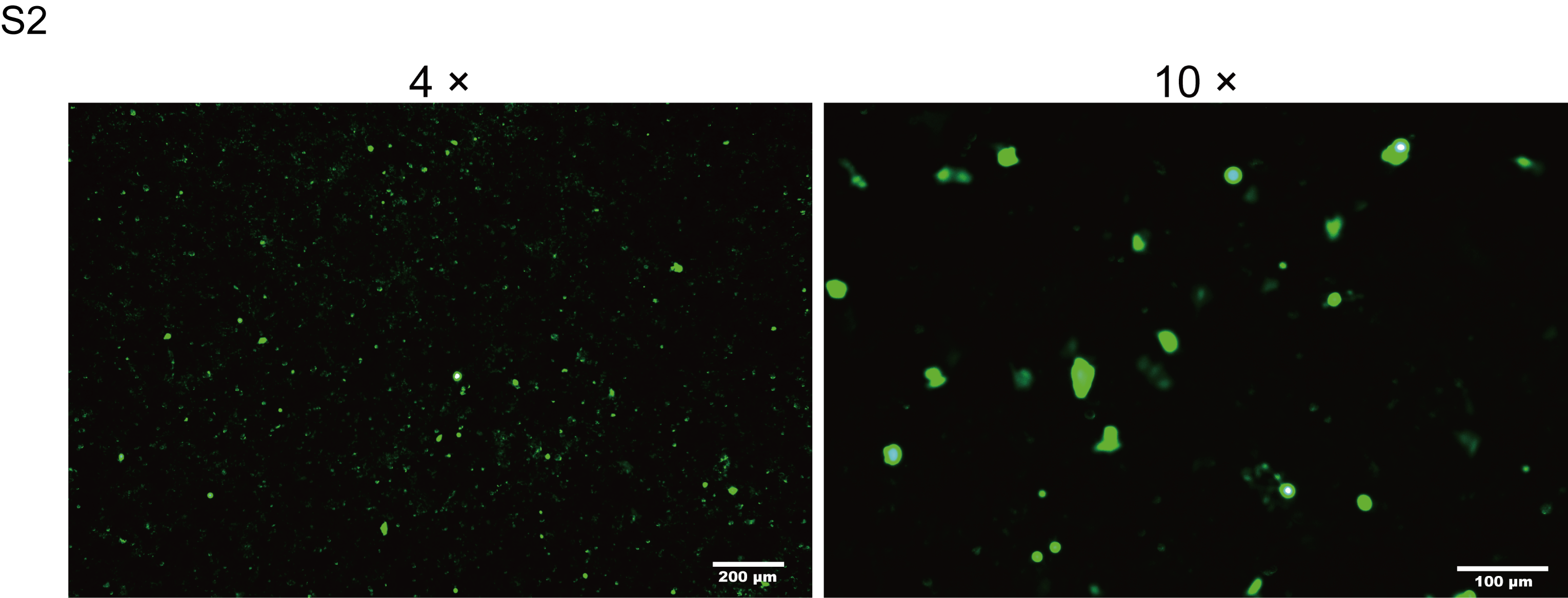

Supplement: Supplementary Figure 2 — Transfect the plasmid carrying EGFP fluorescence into A549 cells and observe the fluorescence under a fluorescence microscope (4×: Scale bar=200 μm and 10×: Scale bar=100 μm). [file Image2.tif]

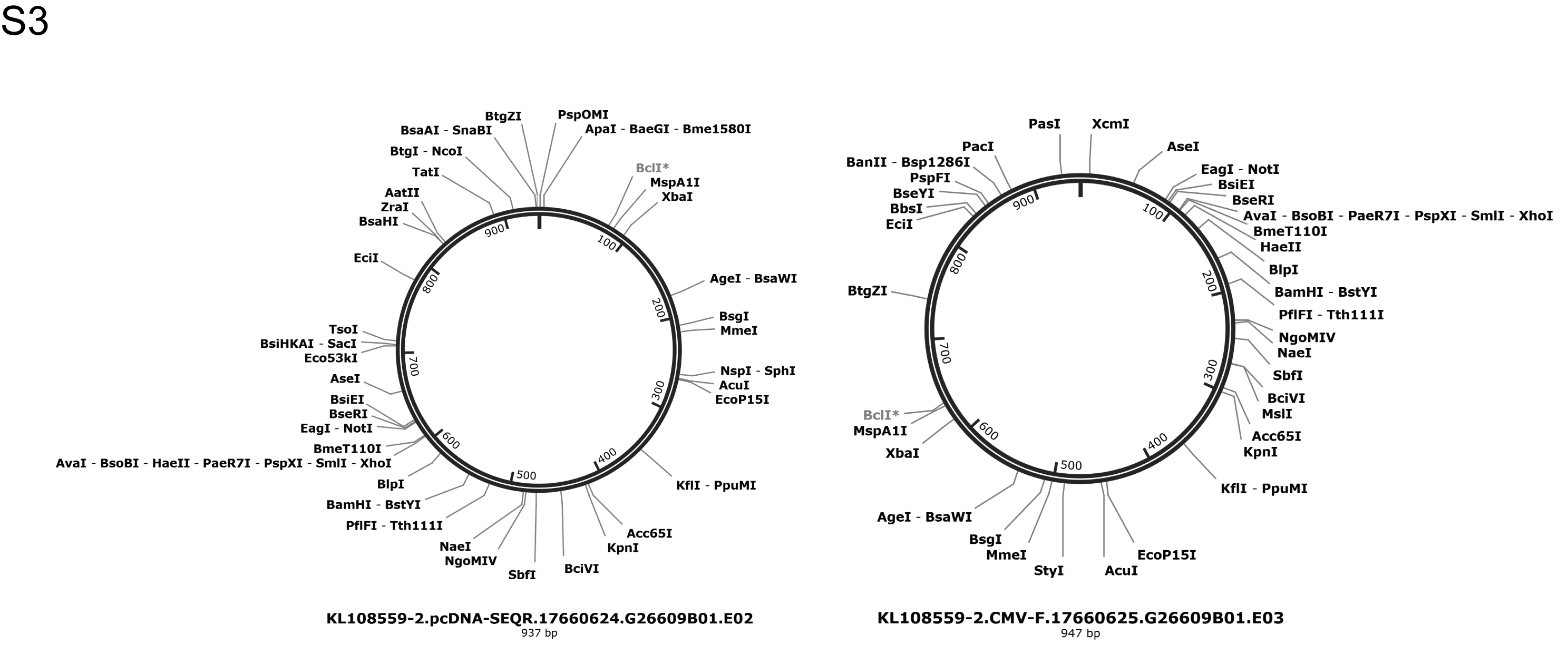

Supplement: Supplementary Figure 3 — Construction of overexpression adenovirus vectors. [file Image3.tif]

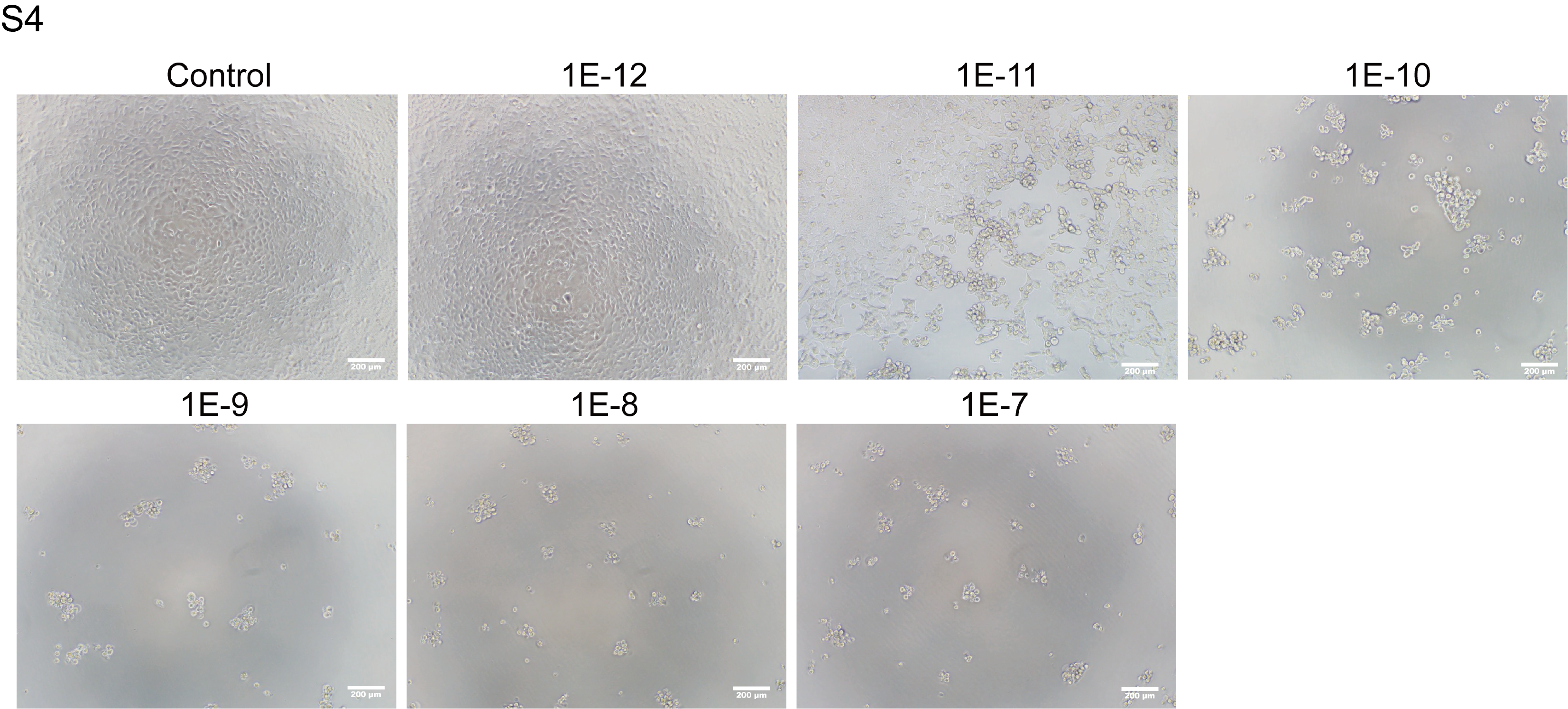

Supplement: Supplementary Figure 4 — End-point dilution method to determine the titer of the virus (4×: Scale bar=200 μm). [file Image4.tif]

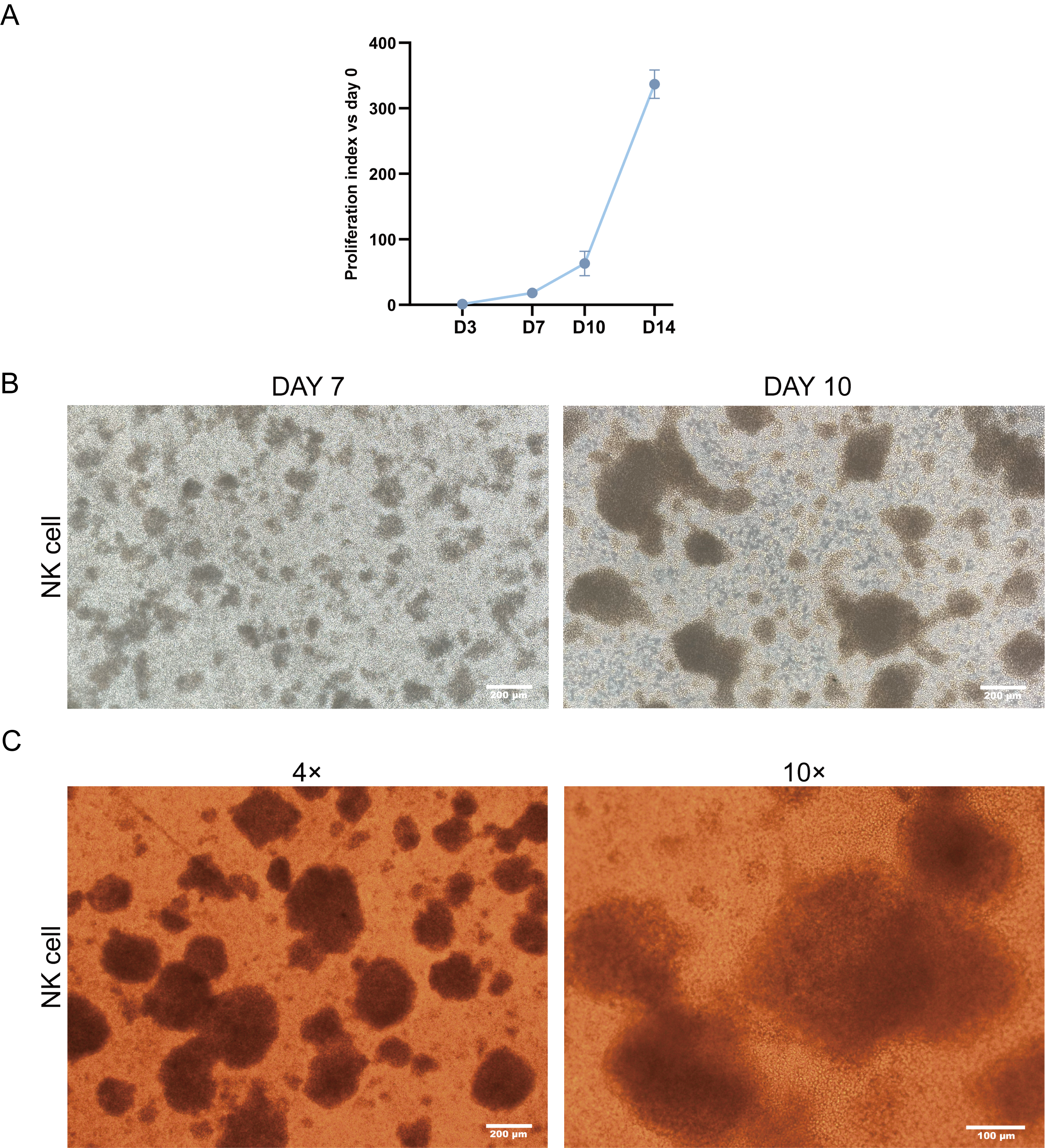

Supplement: Supplementary Figure 5 — The expansion index and morphological characteristics of natural killer (NK) cells. (A) Growth curve of NK cells. (B) Observation of NK cell morphology under a 4× objective lens of an inverted microscope on day 7 and day 10 of in vitro expansion (4×: Scale bar=200 μm). (C) Morphology of NK cells under an inverted microscope on day 14 of in vitro expansion (4×: Scale bar=200 μm and 10×: Scale bar=100 μm). [file Image5.tif]

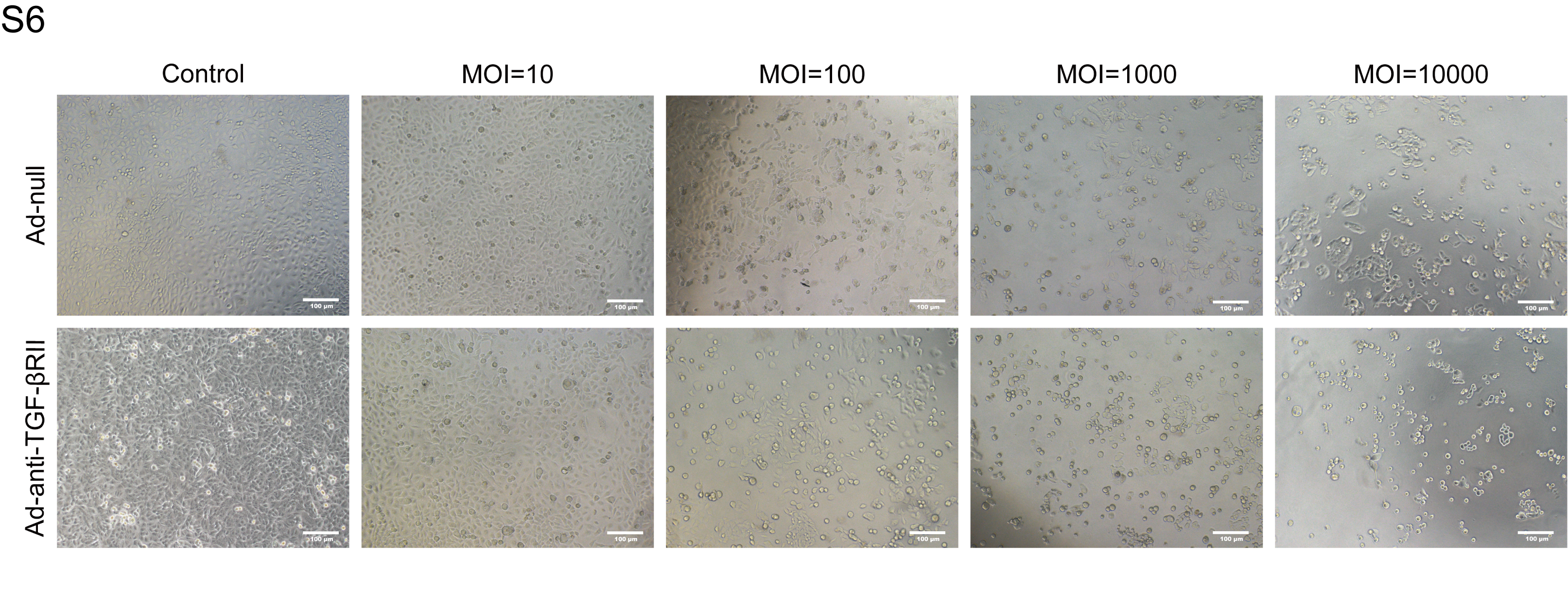

Supplement: Supplementary Figure 6 — A549 cells were infected with Ad-null and Ad-anti-TGF-βRII at MOI of 10, 10, 1000, 10000 for 48 h and then photographed. Pictures were taken 48 h later. Dead or dying cells could be identified by their rounded (4×: Scale bar=200 μm). [file Image6.tif]
